# Accuracy of self-reported height measurements in parents and its effect on mid-parental target height calculation

**DOI:** 10.1186/1472-6823-7-2

**Published:** 2007-04-02

**Authors:** Ieva Braziuniene, Thomas A Wilson, Andrew H Lane

**Affiliations:** 1 Department of Pediatrics, State University of New York, Stony Brook, NY, USA

## Abstract

**Background:**

Clinical determination of mid-parental height is an important part of the assessment of a child's growth, however our clinical impression has been that parents cannot be relied upon to accurately report their own heights. Therefore, we conducted this study to assess the accuracy of parental height self-reporting and its effect on calculated mid-parental target height for children presenting to a pediatric endocrinology office.

**Methods:**

All parents bringing their children for an initial evaluation to a pediatric endocrinology clinic over a period of nine months were questioned and then measured by a pediatric endocrinologist. Parents were blinded to the study. Mid-parental target heights, based on reported and actual height were compared.

**Results:**

There were 241 families: 98 fathers and 217 mothers in our study. Mean measured paternal height was 173.2 cm, self reported 174.9 cm (p < 0.0001), partner reported 177 cm (p = 0.0004). Only 50% of fathers and 58% of mothers reported their height within ± 2 cm of their measured height, while 15% of fathers and 12% of mothers were inaccurate by more than 4 cm. Mean measured maternal height was 160.6 cm, self-reported 161.1 cm (NS), partner reported 161.7 cm (NS). Inaccuracy of height self-report had a small but significant effect on the mean MPTH (0.4 cm, p = 0.045). Analysis showed that only 70% of MPTH calculated by reported heights fell within ± 2 cm of MPTH calculated using measured heights, 24% being in ± 2–4 cm range, and 6% were inaccurate by more than 4 cm.

**Conclusion:**

There is a significant difference in paternal measured versus reported heights with an overall trend for fathers to overestimate their own height. A large subset of parents makes a substantial error in their height self-report, which leads to erroneous MPTH. Inaccuracy is even greater when one parent reports the other parent's height. When a child's growth is in question, measured rather than reported parental heights should be obtained.

## Background

Determination of the mid-parental target height (MPTH) is a critical first step in the assessment of a child with short stature, and obtaining accurate parental heights is essential when determining the MPTH [1-4]. Parental height often determines the extent of a work up and/or therapeutic intervention, and serves as an outcomes benchmark for clinical trials. Our experience has been that primary care physicians usually ask for but infrequently measure parental heights. We have casually observed that often there is a significant discrepancy between reported and measured parental heights. To date several epidemiological studies have been conducted investigating the accuracy of self-reported anthropometric measurements in different population groups [5-12]. These studies revealed that there is a substantial error in individual reports with overall trends to overestimate own height and underestimate own weight. Height overestimation was more prominent among the short adults. Many of the short children referred to a pediatric endocrine clinic come from the short families and if their parents in fact do overestimate their heights, there may be a mismatch between children's actual and expected heights leading to unnecessary worry and investigations.

Both the populations studied and the setting of the previous epidemiological studies does not closely resemble those encountered in pediatric clinical practice, where parents are questioned without them expecting to be measured. On the other hand they have a particular interest in their child's height which might either improve or worsen their own accuracy. Many studies on the outcome of therapeutic interventions to increase final height have compared final height to mid-parental target heights, which are usually obtained in these studies from parental report [13-15].

We designed this study to assess whether there is a significant difference between the reported and measured parental heights from our referral population. We also investigated whether the accuracy of height self reports depended on person's gender, height, ethnic background or the reason for which the child had been brought for the evaluation, and whether any error in height reporting, if such existed, affected the accuracy of MPTH.

## Methods

Over a nine-month period we first questioned and then measured all parents bringing their children for an initial visit to our pediatric endocrinology clinic located within our institution. The study was approved by our institutional review board. All measurements were performed by the pediatric endocrinology attending or a fellow conducting the study using calibrated wall mounted stadiometer (Ayrton Co. USA, model S100). Measurements were made by two investigators (AHL and TAW) whose techniques were compared. There were no significant differences in measurements between these two investigators (unpublished data). At the time of questioning the subjects did not anticipate that their heights would be measured and they were not informed that the data was being collected for the study. If only one parent was present, he or she was asked to estimate their partner's height. Data on subject's age, height and gender, presenting concern (short stature or other); parental reported and actual heights were also recorded.

We compared the mean measured and reported heights and analyzed the variability of error of reported height from the measured and correlation between parental height SDS and accuracy of height self-report. We assessed what effect the discrepancy between measured and reported heights had on mid-parental target height (MPTH), which was calculated according to the formula: (father's height + mother's height + 13 cm)/2 for boys and (father's height + mother's height - 13 cm)/2 for girls, both using reported and measured heights. In the families where only one parent was present, mid-parental target heights were not calculated.

We used the SPSS statistical program to calculate the means, 95% confidence intervals (CI) and standard deviation score (SDS). T-test for independent and paired samples, as appropriate, was used to compare the mean heights in various groups. P value of <0.05 was considered to be statistically significant.

Our Institutional Review Board reviewed the project and determined that informed consent was not required as the consent process would potentially unblind the study and thereby influence the responses of the parents.

## Results

There were 315 subjects from 241 families in our study: 98 fathers and 217 mothers. 101 children were seen for short stature, 140 for other endocrine concerns. Demographic data is summarized in table 1.

**Table 1:** Demographic characteristics

		**Fathers**	**Mothers**	**Total**
**Total in the study**		98	217	315

**Height**	SDS < -1	32	63	95
	SDS -1 to 1	59	129	188
	SDS > 1	7	25	32
**Reason for the visit**				
	Short stature	55	87	130
	Other	43	130	185
**Ethnicity**	White	72	169	239
	Hispanic	17	32	49
	Other	9	15	23

### Fathers

**Figure 1:**
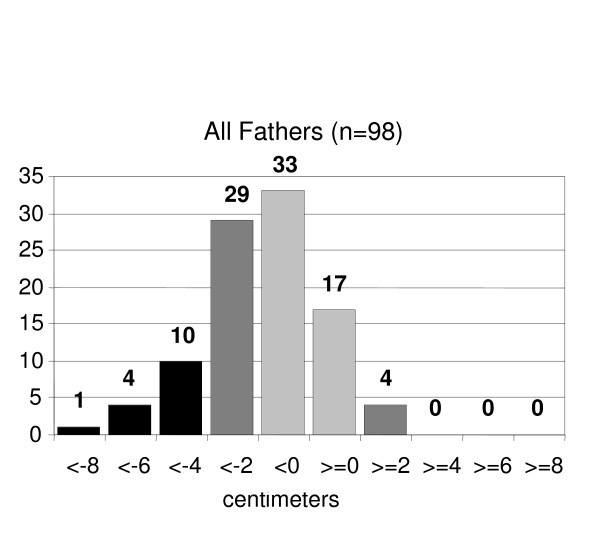
Distribution of accuracy of parental height self-report (actual minus reported heights in centimeters)- fathers.

Mean measured paternal height was 173.2 cm (SD ± 7.0; 95% CI ± 1.4), self reported height was 174.9 cm (SD ± 7.6; 95% CI ± 1.5, p < 0.0001). The fathers' report error (measured height minus reported height) ranged from -8.4 to +3.6 cm. Approximately half of the fathers (50%) reported their height within ± 2 cm of their measured height (figure 1). Mean reported paternal height by the mother was 177 cm (SD ± 8.3; 95% CI ± 1.4) which was significantly greater than the fathers' measured heights (p = 0.0004).

### Mothers

**Figure 2:**
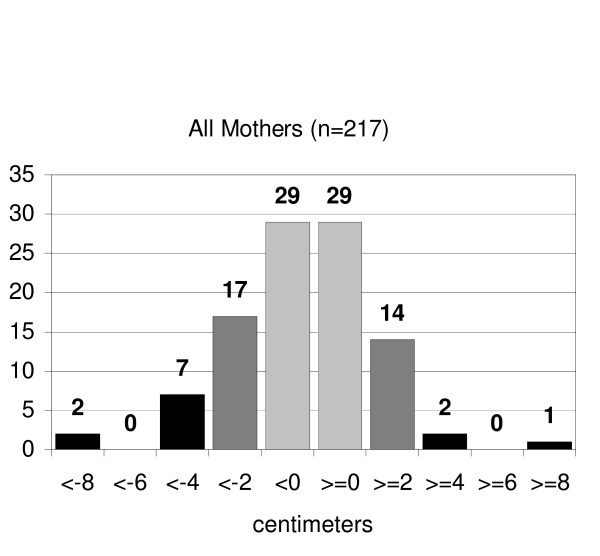
Distribution of accuracy of parental height self-report (actual minus reported heights in centimeters)- mothers.

Mean measured maternal height was 160.6 cm (SD ± 7.3; 95%CI ± 0.97), self-reported 161.1 cm (SD ± 8.1; 95%CI ± 1.09, NS). The mothers' report error (measured height minus reported height) ranged from -11.8 to +22.1 cm. As with the fathers, approximately half (58%) of all mothers reported their heights within ± 2 cm of their measured height (figure 2). Mean reported maternal height by the father was 161.7 cm (SD ± 5.6; 95%CI ± 2.43), which was not significantly greater than the mothers' measured heights.

### Subgroup analysis

**Figure 3:**
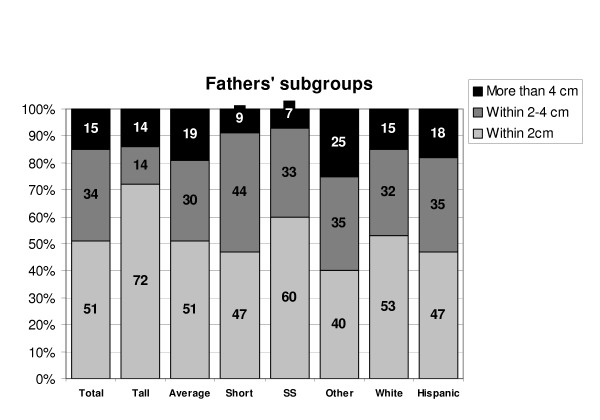
Subgroup analysis – fathers. Percentage of parents in each group who reported their heights accurately, moderately inaccurately and very inaccurately. Total: all groups, Tall: parents with height SDS of >1, Average: SDS -1 to 1, Short: SDS of <0, SS: parents of a child with chief complaint of short stature, Other: parents of a child with non-short stature complaint, White: white parents, Hispanic: Hispanic parents.

Among the fathers there was a trend of more substantial height overestimation in the group of average men than the group of taller or shorter men. The average men had a mean overestimate of being 2.0 cm taller than their measured heights, (range 4.5 cm shorter than measured to 8.4 cm taller) while the tall men had a mean overestimate of being 0.6 cm taller than measured, (range 1.3 cm shorter to 4.2 cm taller) and the short men had a mean overestimate of being 1.3 cm taller than measured (range 3.6 cm shorter to 7.3 cm taller)(figure 3).

**Figure 4:**
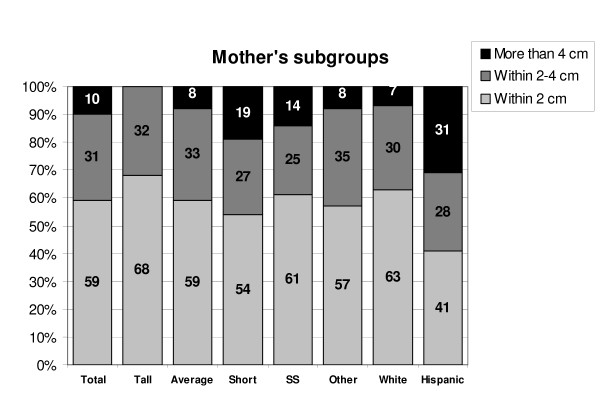
Subgroup analysis – mothers.

Among the mothers, there was no significant correlation between the height and mean reported height, but short women made more frequent erroneous own height assessments and to a larger degree. The short mothers' mean overestimate was 0.4 cm taller than measured (range 22.1 cm shorter to 11.8 cm taller), average mothers' mean overestimate was 0.3 cm taller (range 4.5 cm shorter to 8.2 cm taller) and tall mothers' overestimate was 0.6 cm taller (range 2.6 cm shorter to 3.6 cm taller) (figure 4).

Parents who brought their children for short stature evaluation were more accurate in their height self-report compared to the parents who brought their children for other endocrine concerns.

Among the mothers, Hispanic women had the most variability in their own height judgment (mean overestimate of 1.0 cm taller than measured height, range 22.1 cm shorter to 11.8 cm taller). Forty one percent of their reported heights were in ± 2 cm range and 31% more than 4 cm inaccurate. The Caucasian women were somewhat more accurate with 63% of their reported heights within the ± 2 cm range and 7% more than 4 cm inaccurate. African American and Asian groups had insufficient numbers of parents to be assessed.

Hispanic fathers reported their own heights with a mean overestimate of 1.1 cm taller than the measured heights (range 3.4 cm shorter to 7.2 cm taller), while Caucasian fathers had a mean overestimate of 1.7 cm taller than measured (range 3.6 cm shorter to 7.3 cm taller).

### Effect on mid-parental target height

Mean MPTH calculated using measured parental heights was 165.3 cm (95%CI ± 2.0), and using reported heights 165.7 (95%CI ± 2.2, p = 0.045). Individual error ranged from -7.9 cm to +9.8 cm. Seventy percent of the MPTHs calculated by reported heights fell within ± 2 cm of MPTHs calculated using measured heights, 24% were in ± 2–4 cm range, and 6% were more than by 4 cm inaccurate.

## Discussion

Our study revealed that overall there was a small but significant difference in paternal measured versus reported heights with the trend for fathers to overestimate their own height by a mean of 1.7 cm., which is similar to the results of the previous investigations [5-12]. We did not find a significant difference in mean maternal measured versus reported heights.

We found a substantial individual variability in height self report both among the fathers and the mothers. While most of the parents were reasonably accurate, about 10% of the mothers and 15% of the fathers reported their height with at least 4 cm error. Contrary to our expectations, the men of average (height SDS ± 1) rather than short (height SDS <1) stature overestimated their heights more frequently and to a higher degree. The short mothers were more inaccurate than the tall ones, but there was no significant correlation between mean maternal actual height and the degree of the error. Of note, Hispanic women were over-represented in the short group, and they were the most inaccurate with respect to their actual height, which may have skewed the results in this subgroup. Hispanic fathers demonstrated similar results, but to a smaller degree. It is possible that lack of familiarity with the English measurement system may have contributed to their inaccuracy. We also observed that there was a large discrepancy of 3.8 cm in the mean heights of the fathers, when the fathers whose heights were actually measured were compared to the mean of paternal heights obtained by maternal reports. This underscores the likelihood of clinical error when relying on spousal report of the other partner's height.

A recent study of parents recruited at a non-endocrinology pediatric clinic found a slightly lower mean difference of +1.09 cm for males and -0.09 cm for females. The difference between these results and ours could be because the parents were told of the nature of the study before they were asked about their height and before measurements were taken. In contrast, our parents were unaware that they were taking part in a study. Similar to our study, the authors also reported substantial individual variability[16].

When MPTH's were calculated, most of the inaccuracy was caused by height overestimation, which could erroneously place the child below the normal growth channel. Measuring accurate parental heights and discussing these measurements and calculated MPTH is an important component of educating families presenting with a concern about the child's growth. Taking measurements rather than relying on reported heights may help avoid unnecessary referrals and laboratory investigations of an otherwise normal short child. It has been shown that the perceived rather than actual short stature has a more substantial influence on person's psychosocial functioning[17]; taking measurements may help reduce inaccurate perceptions.

## Conclusion

In conclusion, a large proportion of parents bringing their children to pediatric endocrinology clinic make a significant error reporting their own heights, which has an influence on mid-parental target height calculation. Therefore, if there is a concern about the normality of child's growth, especially if there is a discrepancy between child's expected and actual height, measured rather than reported parental heights should be used. If only one parent is reporting the heights of both it is important to measure the absent parent at a later date if possible. Only the measured heights should be used in clinical studies, especially where a therapeutic outcome such as final height is compared to a pre-treatment calculated MPTH.

## Competing interests

The author(s) declare that they have no competing interests.

## Authors' contributions

IB, TAW, and AHL participated in the design of the study, the collection of data and the authorship and review of the manuscript. All authors read and approved the final manuscript. IB performed the statistical analysis. AHL conceived of the study.

## Pre-publication history

The pre-publication history for this paper can be accessed here:


